# Validating Methods To Eradicate Plant-Pathogenic Ralstonia Strains Reveals that Growth *In Planta* Increases Bacterial Stress Tolerance

**DOI:** 10.1128/spectrum.02270-22

**Published:** 2022-12-01

**Authors:** Madeline M. Hayes, Ronnie J. Dewberry, Lavanya Babujee, Rebecca Moritz, Caitilyn Allen

**Affiliations:** a Department of Plant Pathology, University of Wisconsin—Madison, Madison, Wisconsin, USA; b Select Agent Program, Environment, Health, and Safety, University of Wisconsin—Madison, Madison, Wisconsin, USA; Pennsylvania State University

**Keywords:** select agent, eradication, stress tolerance, *Ralstonia solanacearum* race 3 biovar 2, bacterial wilt, potato brown rot, Southern wilt of geranium

## Abstract

Plant-pathogenic bacteria in the Ralstonia solanacearum species complex (RSSC) cause highly destructive bacterial wilt disease of diverse crops. Wilt disease prevention and management is difficult because RSSC persists in soil, water, and plant material. Growers need practical methods to kill these pathogens in irrigation water, a common source of disease outbreaks. Additionally, the R. solanacearum race 3 biovar 2 (R3bv2) subgroup is a quarantine pest in many countries and a highly regulated select agent pathogen in the United States. Plant protection officials and researchers need validated protocols to eradicate R3bv2 for regulatory compliance. To meet these needs, we measured the survival of four R3bv2 and three phylotype I RSSC strains following treatment with hydrogen peroxide, stabilized hydrogen peroxide (Huwa-San), active chlorine, heat, UV radiation, and desiccation. No surviving RSSC cells were detected after cultured bacteria were exposed for 10 min to 400 ppm hydrogen peroxide, 50 ppm Huwa-San, 50 ppm active chlorine, or temperatures above 50°C. RSSC cells on agar plates were eradicated by 30 s of UV irradiation and killed by desiccation on most biotic and all abiotic surfaces tested. RSSC bacteria did not survive the cell lysis steps of four nucleic acid extraction protocols. However, bacteria *in planta* were more difficult to kill. Stems of infected tomato plants contained a subpopulation of bacteria with increased tolerance of heat and UV light, but not oxidative stress. This result has significant management implications. We demonstrate the utility of these protocols for compliance with select agent research regulations and for management of a bacterial wilt outbreak in the field.

**IMPORTANCE** Bacteria in the Ralstonia solanacearum species complex (RSSC) are globally distributed and cause destructive vascular wilt diseases of many high-value crops. These aggressive pathogens spread in diseased plant material and via contaminated soil, tools, and irrigation water. A subgroup of the RSSC, race 3 biovar 2, is a European and Canadian quarantine pathogen and a U.S. select agent subject to stringent and constantly evolving regulations intended to prevent pathogen introduction or release. We validated eradication and inactivation methods that can be used by (i) growers seeking to disinfest water and manage bacterial wilt disease outbreaks, (ii) researchers who must remain in compliance with regulations, and (iii) regulators who are expected to define containment practices. Relevant to all these stakeholders, we show that while cultured RSSC cells are sensitive to relatively low levels of oxidative chemicals, desiccation, and heat, more aggressive treatment, such as autoclaving or incineration, is required to eradicate plant-pathogenic Ralstonia growing inside plant material.

## INTRODUCTION

Plant-pathogenic bacteria in the Ralstonia solanacearum species complex (RSSC) are globally distributed and highly destructive. Collectively, this genetically diverse species complex can cause bacterial wilt disease in over 250 plant hosts, including high-value agricultural exports and key subsistence crops like potato, tomato, and banana (1). Plant-pathogenic Ralstonia are transmitted via contaminated plant matter, soil, and water, making them very difficult to eradicate once they are established.

Though the bacteria in the RSSC are usually considered tropical pathogens, a small subset of strains cause potato brown rot at temperatures as low as 20°C in tropical highlands and temperate zones (2). These strains fall into the phylotype II sequevar 1 subgroup, which is known historically and for regulatory purposes as R. solanacearum race 3 biovar 2 (R3Bv2) (3). Potato brown rot, which can cause up to 100% losses and is among the most destructive diseases of potatoes, can be disseminated via latently infected seed potato tubers and ornamental cuttings (4, 5). Regulatory agencies hope to prevent the spread of potato brown rot in higher latitudes, so R. solanacearum R3bv2 is listed as a high-concern quarantine pest in Canada and Europe and is a select agent (SA) pathogen in the United States (6).

The U.S. select agent list includes microbes considered potential bioterrorism threats to humans, livestock, or crops. SA regulations evolve continuously to manage threats of pathogen release from accidental error, inadequate decontamination, or security flaws. For example, following an accidental release of live anthrax spores from an SA laboratory, SA regulations now require validated protocols that ensure all select agents are biologically inactivated before downstream applications like experiments with extracted proteins or nucleic acids (7). Institutional SA programs throughout the United States work closely with research laboratories to identify such applications and ensure that each inactivation protocol is appropriately validated.

Standardized inactivation protocols can prevent the spread of pathogens, keep research laboratories in regulatory compliance, and help growers manage disease. However, a literature survey found no validated chemical and physical treatment methods to eliminate plant-pathogenic Ralstonia from cultures, contaminated soil, water, or plant material.

To address this need, we defined treatments that will inactivate plant-pathogenic Ralstonia cells under laboratory and agricultural conditions. We identified the levels of bleach (active Cl^−^), oxidative stress, UV radiation, heat, and desiccation required to eradicate plant-pathogenic Ralstonia cells from water, plant matter, and laboratory surfaces. Treatments were validated on seven RSSC strains, including four R3bv2 SA strains and three other RSSC strains isolated from recent bacterial wilt outbreaks. Additionally, we validated that no viable plant-pathogenic Ralstonia cells survived four protocols for extraction of nucleic acids, which are commonly used for downstream research and diagnostic purposes. There were no differences in treatment efficacy between SA and non-SA strains. However, a subpopulation of plant-pathogenic Ralstonia cells growing in plants were highly resistant to heat and UV radiation. This finding has significant implications for eradication protocols and effective bacterial wilt disease management.

## RESULTS

To determine the limits of survival for diverse plant-pathogenic Ralstonia strains under field and laboratory conditions, we identified the lethal doses of various chemical and physical treatments. The 100% lethal dose (LD_100_) was defined as the treatment necessary to reduce surviving cells below our 10-CFU/mL limit of detection. We tested treatments on 10^10^-CFU/mL cell suspensions because such dense cultures are common in the laboratory. This is much higher than the 10^2^ to 10^5^ CFU/g observed in naturally infested field soils or water, but any treatment effective at 10^10^ CFU/mL was also effective at lower densities (data not shown). Plant-pathogenic Ralstonia cells in infected plants were subjected to eradication treatments when wilt symptoms first appeared, as would happen in the field when growers try to control disease.

### Cultured R. solanacearum cells are sensitive to antimicrobial oxidants.

**FIG 1:**
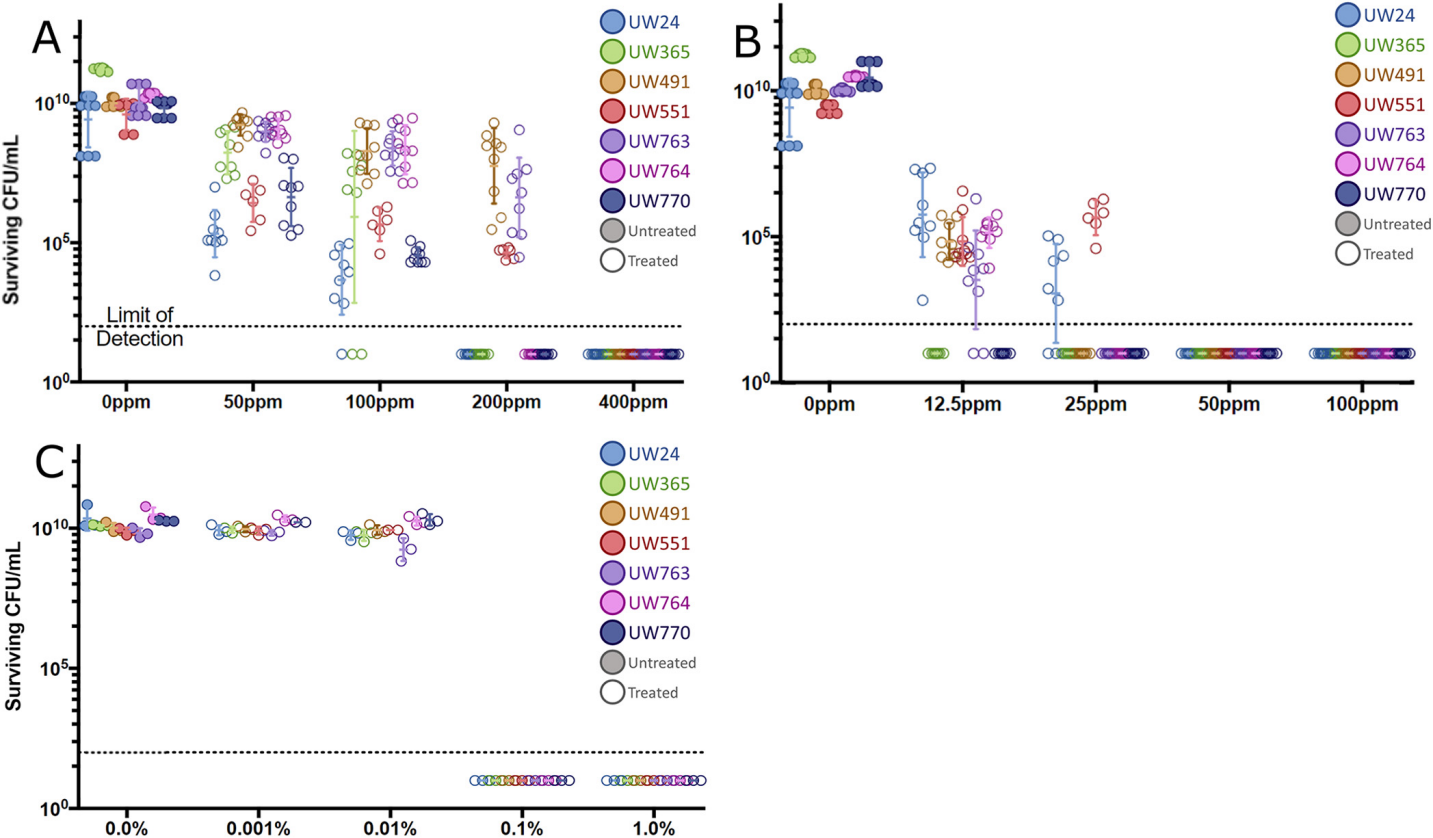
Oxidant concentrations required to eradicate plant-pathogenic Ralstonia cells. Population sizes of seven Ralstonia strains following exposure to antimicrobial oxidants are indicated by colored symbols as indicated in the keys, with untreated samples indicated by filled circles and treated samples indicated by open circles. Each symbol represents the result of one independent experiment, each including three technical replicates. Surviving cells were quantified by serial dilution plating after a 10^10^-CFU/mL bacterial cell suspension in water was incubated for 10 min at room temperature with laboratory grade H_2_O_2_ (A), 50% Huwa-San solution (B), or sodium hypochlorite (C) at the concentrations indicated below. (A) H_2_O_2_, *n* = 9 experiments per strain, Mann-Whitney test was performed on the untreated control group (all replicates, all strains) and the 400-ppm treatment (all replicates, all strains); *P* < 0.001. (B) Huwa-San, *n* = 9 experiments per strain, Mann-Whitney test was performed on the untreated control group (all replicates, all strains) and the 50-ppm treatment (all replicates, all strains); *P* < 0.001. (C) Sodium hypochlorite, *n* = 3 experiments per strain, Mann-Whitney test was performed on the untreated control group (all replicates, all strains) and the 0.1% (vol/vol) treatment (all replicates, all strains); *P < *0.001.

We measured the ability of three antimicrobial chemicals, hydrogen peroxide, Huwa-San, and bleach, to kill cultured plant-pathogenic Ralstonia cells in water. A 10-min exposure to 400 ppm hydrogen peroxide reduced the populations of all tested strains to below the limit of detection (Fig. 1A). Strains UW24, UW365, UW764, and UW770 did not survive 200 ppm hydrogen peroxide for 10 min. Treating bacteria with 50 ppm Huwa-San, a chemically stabilized form of hydrogen peroxide, also left no detectable CFU of any strain (Fig. 1B). Some strains were more sensitive to Huwa-San: 25 ppm eliminated UW491, UW763, and UW764 and only 12.5 ppm left no detectable CFU of strains UW365 and UW770. Active chlorine was highly effective against plant-pathogenic Ralstonia. A 10-min exposure to 0.1% (vol/vol) commercial bleach solution (8.25% sodium hypochlorite) in water, equivalent to 120 μM active chloride ion, left no detectable cells of all tested plant-pathogenic Ralstonia strains (Fig. 1C). In summary, relatively low concentrations of these oxidative stress-inducing chemicals killed plant-pathogenic Ralstonia. The LD_100_ values of these antimicrobial chemicals were not affected by culture growth phase, showing similar efficacies against log-phase and stationary-phase cells (data not shown).

### Efficacy of physical treatments against plant-pathogenic *Ralstonia*.

**FIG 2:**
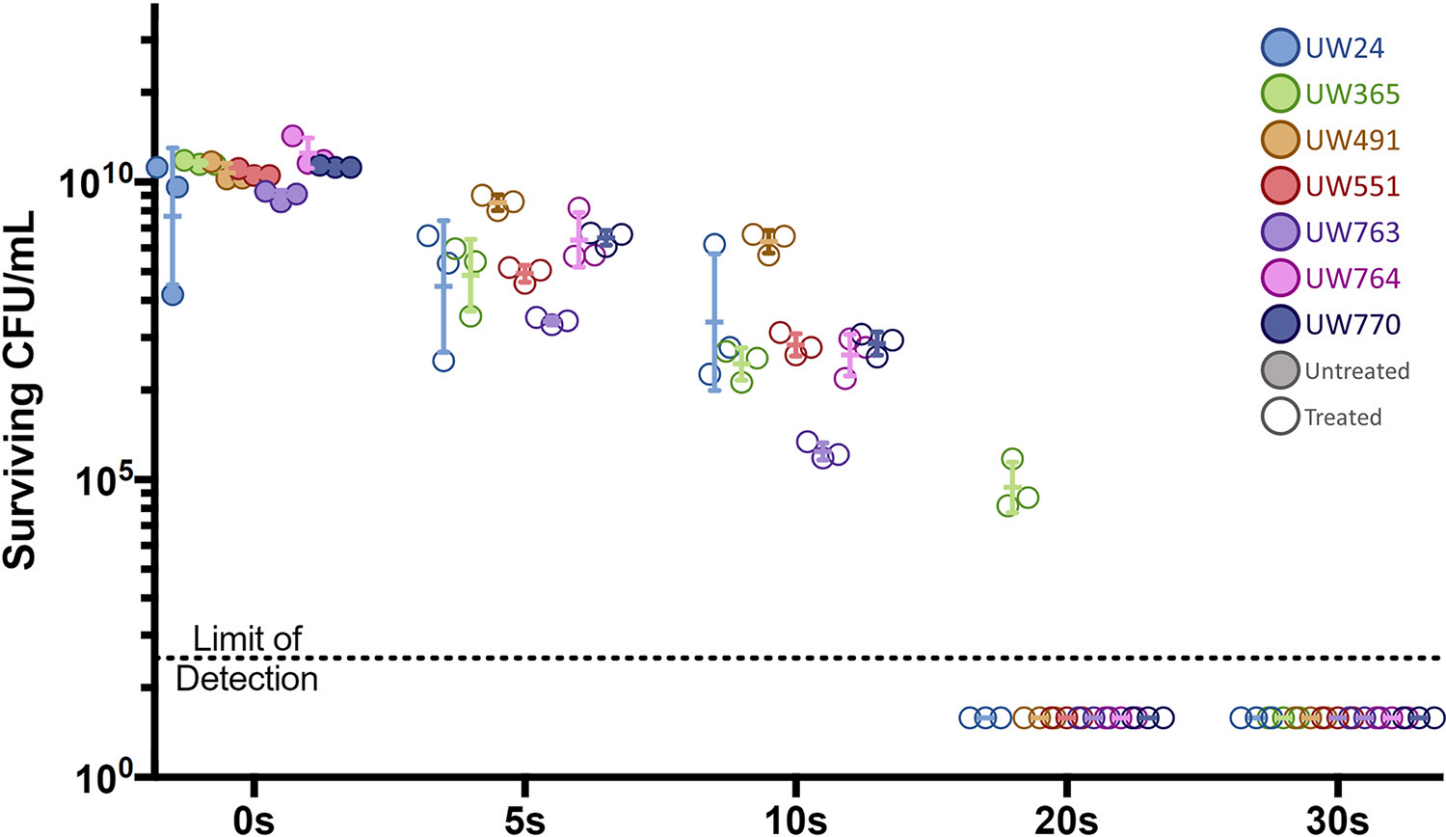
UV radiation required to eradicate plant-pathogenic Ralstonia cells from surfaces. Population sizes of seven plant-pathogenic Ralstonia strains following exposure to 0.2 μmol/m^2^/s UV light on CPG agar plates for the times indicated on the *x* axis are shown by colored symbols as detailed in the key, with untreated samples indicated by filled circles and treated samples indicated by open circles. Surviving cells were quantified by counting colonies that grew after treatment. Each symbol represents one independent experiment with three technical replicates (*n* = 3 experiments per strain). Mann-Whitney test was performed on the untreated control group (all replicates, all strains) and the 30-s treatment (all replicates, all strains); *P* < 0.001.

UV radiation was effective against cultured bacteria spread on agar plates. Irradiation for 20 s reduced survival to below the limit of detection of all plant-pathogenic Ralstonia strains except UW365, which required 30 s of exposure (Fig. 2). This indicates that germicidal UV radiation produced by a standard biological safety cabinet can quickly decontaminate surfaces infested by plant-pathogenic Ralstonia.

Desiccation is not usually a practical decontamination procedure, but it is useful to know a pathogen’s desiccation tolerance when cleaning growth facilities or disposing of contaminated materials. We asked if a dry paper towel or other porous fomite (surface) can harbor viable pathogen cells and pose a risk for release. We measured bacterial survival after plant-pathogenic Ralstonia cell suspensions were dried at 28°C for 120 h in open microcentrifuge tubes and then cultured in rich broth. None of the seven tested strains survived this treatment. Similarly, none of the strains survived 120 h of desiccation on sterile laboratory paper towels, indicating that cultured Ralstonia cells are intolerant of desiccation (Table 1). This finding suggests that fabric, such as lab coats, contaminated with Ralstonia cells can be effectively disinfested with 120 h in a drying oven.

**TABLE 1:** Desiccation on surfaces and in plant tissue and soil eradicates plant-pathogenic Ralstonia cells

Strain^*a*^	No. of technical replicates showing survival/total no. of technical replicates tested (*n* = no. of biological replicates)			
	*In planta* ^*b*^	In soil^*c*^	In tubes^*d*^	On fomite^*e*^
UW 24	0/14 (*n* = 3)	NT	0/3 (*n* = 3)	0/9 (*n* = 3)
UW 365	0/13 (*n* = 3)	NT	0/3 (*n* = 3)	0/9 (*n* = 3)
UW 491	0/8 (*n* = 2)	NT	0/3 (*n* = 3)	0/9 (*n* = 3)
UW 551	1/28 (*n* = 3)	0/6 (*n* = 3)	0/3 (*n* = 3)	0/9 (*n* = 3)
UW 763	1/19 (*n* = 3)	NT	0/3 (*n* = 3)	0/9 (*n* = 3)
UW 764	0/17 (*n* = 2)	NT	0/3 (*n* = 3)	0/9 (*n* = 3)
UW 770	0/22 (*n* = 3)	NT	0/3 (*n* = 3)	0/9 (*n* = 3)

a See Table 3 for strain details.

b Infected plant stems were dehydrated at 35°C for 12 h and then used to inoculate SMSA liquid medium, enrichment cultured at 28°C for 48 h, and then qualitatively assessed for bacterial growth as described in Materials and Methods.

c Soil from pots of completely wilted plants was dehydrated and then assessed for survival as with infected plant stems as described in Materials and Methods. NT, not tested.

d Cell suspensions in water (aliquoted into 1.5-mL tubes) were allowed to desiccate at 28°C for 120 h and then used to inoculate CPG liquid medium, enrichment cultured at 28°C for 48 h, and then qualitatively assessed for growth.

e Cell suspensions in water were dropped onto paper towels and allowed to desiccate and then assessed for survival with enrichment culture as with suspensions in tubes described in Materials and Methods.

**FIG 3:**
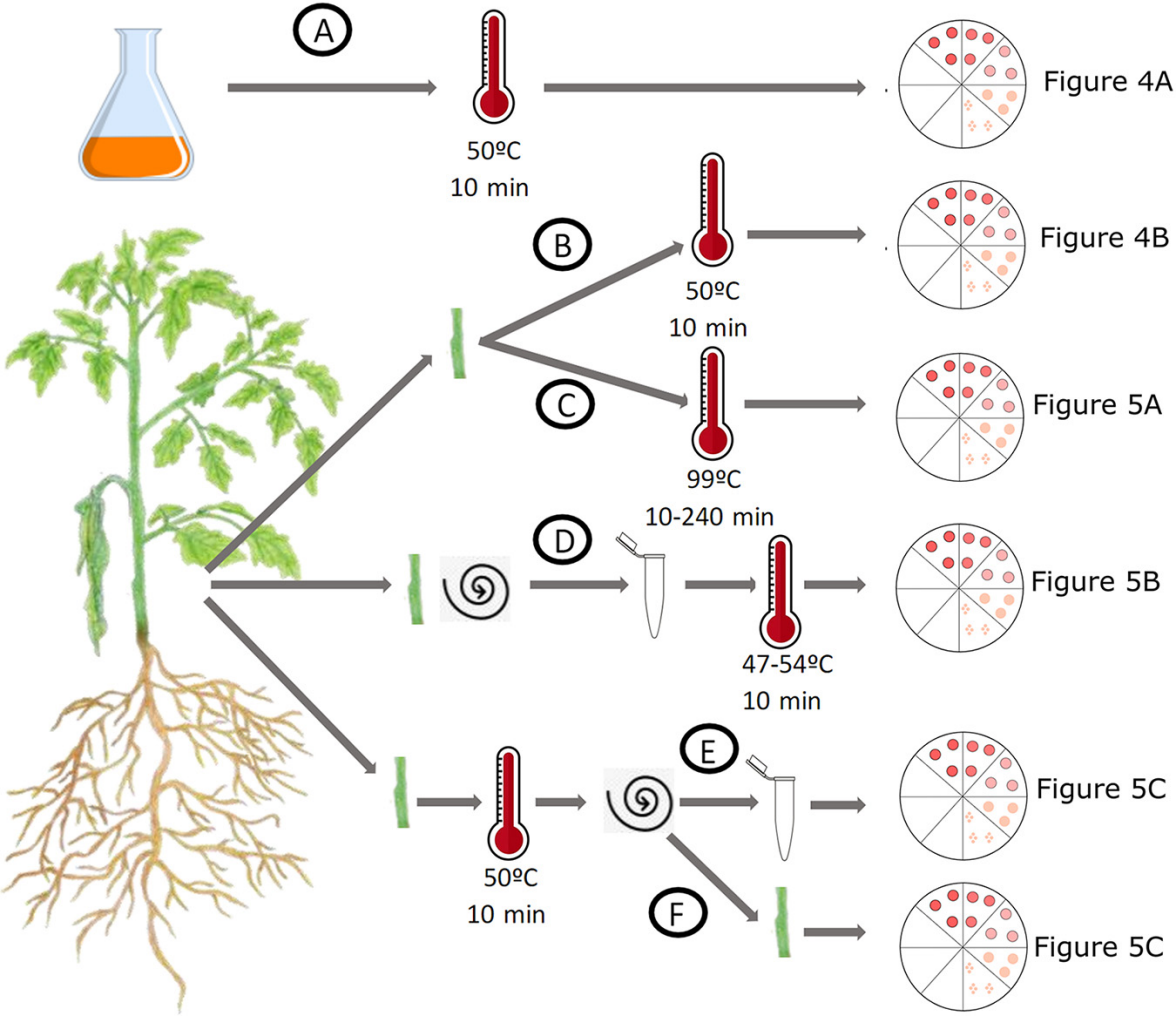
Overview of experiments to determine heat tolerance of plant-pathogenic Ralstonia cells in culture and from infected plants. For all treatments, surviving cells were quantified by serial dilution plating combined with a qualitative enrichment culture to check for surviving cells below the 100-CFU detection limit of dilution plating. Quantitative results for each experiment are displayed as described below. (A) Surviving plant-pathogenic Ralstonia cells after 100 μL of a 10^8^-CFU/mL suspension of cultured cells in a 0.2-mL PCR tube was exposed to 50°C for 10 min. (B) Surviving plant-pathogenic Ralstonia cells after stem sections from infected tomato plants were exposed to 50°C for 10 min. (C) Surviving plant-pathogenic Ralstonia cells after stem sections from infected plants were exposed to 99°C for 10 to 240 min. (D) Surviving cells after planktonic plant-pathogenic Ralstonia cells were extracted from infected tomato stems by gentle centrifugation and then exposed to 47 to 54°C for 10 min. (E, F) Surviving plant-pathogenic Ralstonia cells after stem sections from infected plant tissue were treated at 50°C for 10 min and then gently centrifuged to extract planktonic cells. Both the surviving extracted cells (E) and surviving cells in the ground plant stems (F) were quantified.

**FIG 4:**
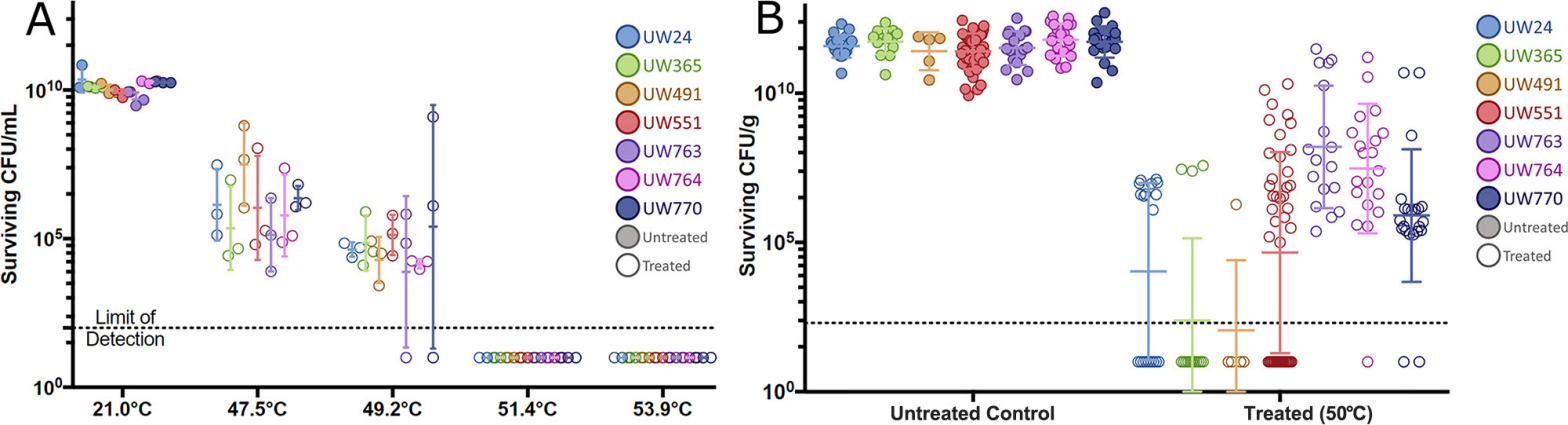
Heat treatments required to eradicate plant-pathogenic Ralstonia cells from culture or from infected plants. (A) Cultured plant-pathogenic Ralstonia cells are very susceptible to heat. Population sizes of seven plant-pathogenic Ralstonia strains following exposure of suspensions of cultured cells in water at the indicated temperatures for 10 min are shown as colored circles as indicated in the key. Untreated samples are indicated by filled circles, and treated samples are indicated by open circles. Surviving cells were quantified by serial dilution plating. *n* = 3 experiments per strain. Mann-Whitney test was performed on the untreated control group (all replicates, all strains) and the 51.4°C treatment (all replicates, all strains); *P* < 0.001. (B) Plant-pathogenic Ralstonia cells in infected plant tissue are more resilient to heat stress. Population sizes of seven strains (indicated by colored symbols) following treatment of stems from infected tomato plants at 50°C for 10 min. Surviving cells were quantified by serial dilution plating. *n* = 2 or 3 biological replicates per strain, 142 technical replicates total. Mann-Whitney signed-rank test was performed on the untreated control group (all replicates, all strains) and the 50°C treatment (all replicates, all strains); *P < *0.0001.

Heat, usually autoclaving, is a common laboratory decontamination procedure. We measured the heat tolerance of plant-pathogenic Ralstonia cells grown under various conditions, as summarized in Fig. 3. We determined that 10 min of exposure to 50°C is the minimum kill temperature for a water suspension of cultured cells of any tested strain (Fig. 4A). This exact kill point was identified using a heat gradient that left surviving cells at 49.2°C but none at 51.4°C (Fig. 4A). Thus, cultured plant-pathogenic Ralstonia cells are efficiently killed by brief exposure to relatively low temperatures.

### Plant-pathogenic *Ralstonia* cells recovered from diseased plant tissue have increased stress tolerance.

**FIG 5:**
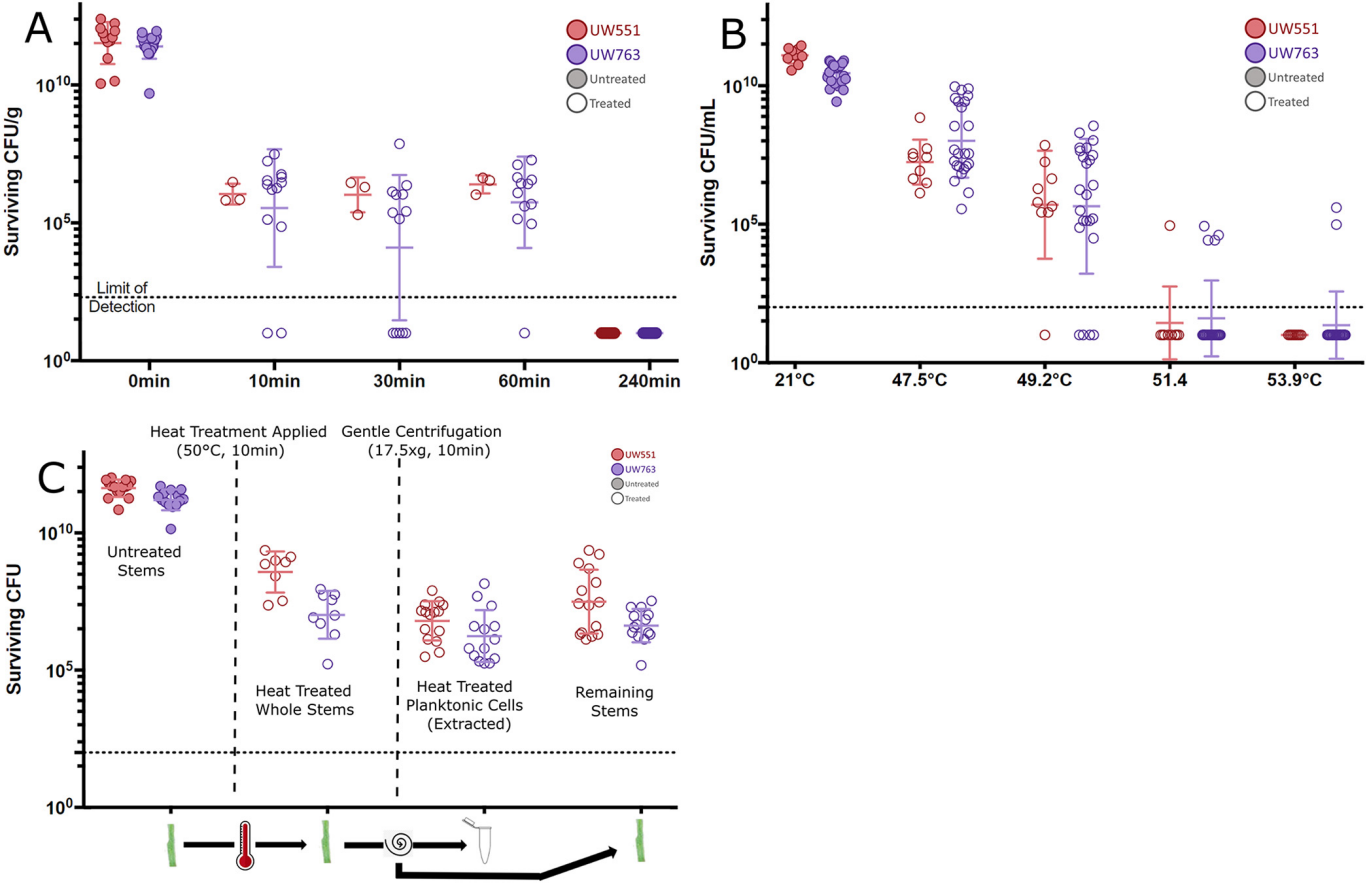
Host-conditioned plant-pathogenic Ralstonia cells have increased heat tolerance, but 99°C eradicated plant-pathogenic Ralstonia cells from plant stems. (A) Population sizes of two plant-pathogenic Ralstonia strains following treatment of infected tomato stems at 99°C for the indicated times are indicated by colored symbols, with untreated samples shown as filled circles and treated samples indicated by open circles. Surviving cells were quantified by dilution plating. *n* = 3 biological replicates per strain. Mann-Whitney test was performed on the untreated control group (all replicates, all strains) and the 240-min treatment (all replicates, all strains); *P < *0.001. (B) A small subpopulation of host-conditioned planktonic cells have modestly higher heat tolerance. Population sizes of two Ralstonia strains (indicated by colored symbols) following extraction from plant stems and treatment for 10 min at the indicated temperatures. *n* = 3 biological replicates per strain. Mann-Whitney test was performed on the untreated control group (all replicates, all strains) and the 51.4°C treatment (all replicates, all strains); *P* value was not significant. (C) Host-conditioned planktonic cells heat treated before extraction from plant tissue show increased heat tolerance. Population sizes of two plant-pathogenic Ralstonia strains (indicated by colored symbols) from left to right: untreated control stems, heat-treated whole stems before planktonic-cell extraction (CFU/g), extracted planktonic cells (CFU/mL), and remaining stems after heat treatment and planktonic-cell extraction (CFU/g). *n* = 3 biological replicates per strain with 14 or 15 technical replicates total. No statistical tests were performed as all replicates showed survival above the limit of detection.

Bacteria growing inside an infected host can differ significantly in physiology and stress tolerance from cells of the same strain growing in culture (8–10), so we validated decontamination procedures on host-conditioned plant-pathogenic Ralstonia cells growing in infected tomato plants, a potential source of the pathogen. We heat treated stem sections harvested from symptomatic plants that had been inoculated with each of the seven Ralstonia strains. These stem sections contained around 10^10^ CFU/g tissue as determined by serial dilution plating. Interestingly, about 0.001% of Ralstonia cells inside tomato stems survived treatment of 10 min at 50°C; this was true of all seven strains tested (Fig. 4B). Similar small populations survived stepwise increases in temperature and exposure time. All Ralstonia cells were killed only after infected stems were incubated for 4 h at 99°C, with no differences among strains (Fig. 5A and data not shown). These results demonstrate that when this pathogen grows in host plants, it has dramatically increased heat tolerance.

To further explore how growth of plant-pathogenic Ralstonia *in planta* increased heat stress tolerance, we focused on two representative strains, phylotype II R3bv2 strain UW551 and phylotype I tomato isolate UW763. Populations of plant-pathogenic Ralstonia inside wilting plants are heterogenous; some planktonic cells float or swim freely in the flowing xylem sap, while others live in dense biofilms along xylem vessel walls (11, 12). We hypothesized that either (i) bacteria growing in tomato xylem are protected from heat stress by the plant tissue itself or (ii) Ralstonia cells in plants are more heat tolerant because their physiology is altered by the host environment, because their physiology is altered by growth in biofilms, or because they are physically protected by the biofilm matrix.

To test the plant protection and host-altered physiology hypotheses, we measured the heat tolerance of planktonic plant-pathogenic Ralstonia cells following their removal from infected tomato stem sections by gentle centrifugation. Centrifuging infected tomato stems extracts only about 10% of the total bacterial population (10). If the plant tissue itself offers physical or chemical protection from heat stress, we would not expect extracted planktonic bacteria to survive for 10 min at 50°C. However, although most planktonic samples from plants were as heat sensitive as Ralstonia grown in culture, 5 of the 33 stems tested contained bacteria with greater heat tolerance, with 15% of replicates across both strains surviving at 51.4°C and two of those surviving at 53.9°C (Fig. 5B). This result could reflect heat-tolerant cells from a biofilm fragment dislodged during centrifugation, or it could indicate that planktonic Ralstonia cells in plants contain a subpopulation of heat-tolerant cells. To further investigate, we first heat treated infected stems for 10 min at 50°C, centrifuged them to remove planktonic cells, and quantified the surviving cells in both the planktonic and stem-associated samples. If the plant offered protection from heat stress, we would expect many planktonic cells to survive the treatment. Indeed, we found that some planktonic cells from inside plant tissue survived 10 min of exposure to 50°C (Fig. 5C). This supports the hypothesis that the plant offers some protection from heat stress, although it is also possible that heat treatment loosened biofilms inside the stems, making it more likely that biofilm fragments were extracted by centrifugation.

To separate the protective effects of growth *in planta* from the protective effects of growing in a biofilm, we measured the stress tolerance of plant-pathogenic Ralstonia biofilms grown on glass slides. If growth in plant tissue protects Ralstonia cells from stress or selects for stress-tolerant cells, then biofilms grown on abiotic glass slides would be no more stress tolerant than cells grown in shaking liquid medium. However, enrichment culture of biofilms on slides after heat treatment revealed that at least some cells in biofilms grown on glass slides also survived treatment at 50°C for 10 min (data not shown). This indicated that growth in biofilms on either biotic or abiotic surfaces increased the heat tolerance of plant-pathogenic Ralstonia.

Because biofilms can protect microbes from diverse stresses, we hypothesized that plant-pathogenic Ralstonia cells living in biofilms in infected plants might also have increased tolerance of UV light, hydrogen peroxide, and Huwa-San but that planktonic cells in xylem sap of diseased plants would have stress tolerance levels similar to those of bacteria grown in broth culture. We tested this hypothesis by subjecting planktonic Ralstonia cells harvested from xylem and resuspended in water at high densities to the physical and chemical antimicrobial treatments described above.

**FIG 6:**
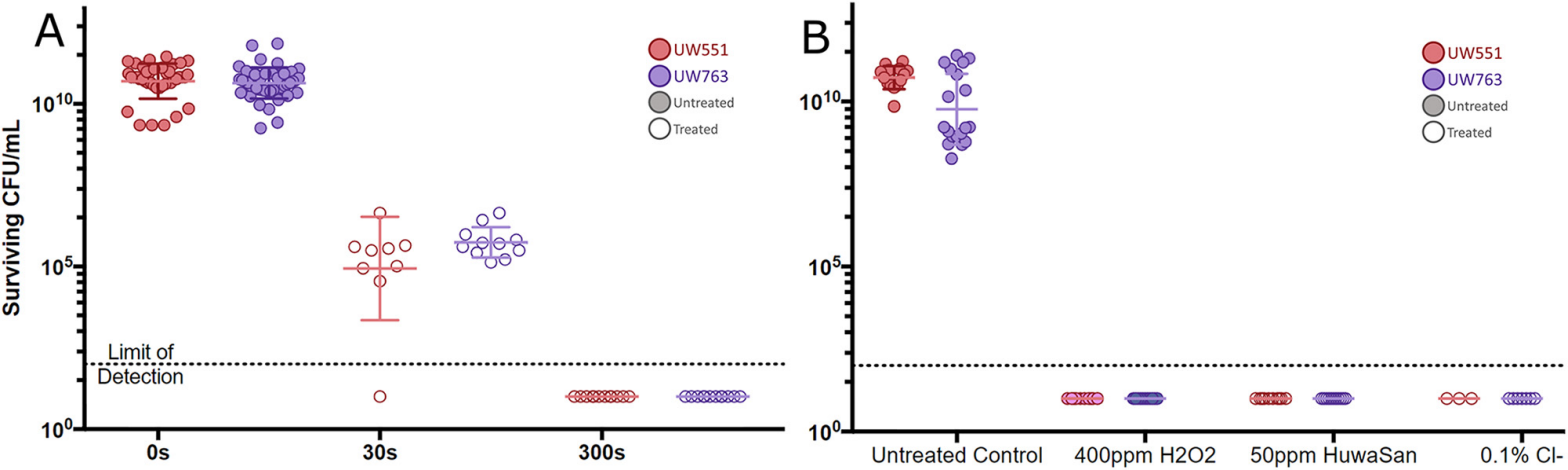
Plant host-conditioned plant-pathogenic Ralstonia cells have increased UV tolerance, but not increased ROS tolerance. (A) Host-conditioned planktonic cells show increased UV tolerance. Population sizes of two Ralstonia strains following extraction from plant stems and exposure to 0.2 μmol/m^2^/s UV light on CPG agar plates for the indicated times are indicated by colored symbols, with untreated samples indicated by filled circles and treated samples indicated by open circles. Surviving cells were quantified by counting colonies that grew after treatment. Mann-Whitney test was performed on the untreated control group (all replicates, all strains) and the 300-s treatment (all replicates, all strains); *P < *0.001. (B) Host-conditioned planktonic cells do not show increased ROS tolerance. Population sizes of two Ralstonia strains (indicated by colored symbols) following extraction from plant stems and treatment for 10 min as indicated. *n* = 3 to 7 biological replicates per strain. Mann-Whitney test was performed on the untreated control group (all replicates, all strains) and the 400-ppm/50-ppm/0.1% (vol/vol) treatments, respectively (all replicates, all strains); *P* < 0.001 for all comparisons.

Consistent with our finding that some Ralstonia cells growing *in planta* have increased heat tolerance, bacteria centrifuged from diseased tomato stems were much more resistant to UV radiation than cultured cells, requiring 5 min (300 s) of exposure to reduce surviving CFU to below the limit of detection, compared to just 30 s for cultured cells (Fig. 6A). These results indicate that plant-pathogenic Ralstonia cells grown in diseased plants have increased stress tolerance even after the bacteria are physically separated from the plant.

In response to pathogen infection, plants produce reactive oxygen species (ROS). Ralstonia cells experience oxidative stress inside host plants (13). To determine whether growth inside a plant host increased the ROS tolerance of plant-pathogenic Ralstonia, we treated planktonic cells centrifuged from diseased tomato stems with the LD_100_ of bleach, hydrogen peroxide, and Huwa-San as determined for cultured cells. Planktonic bacteria from diseased tomato stems were no more tolerant of these three ROS-generating chemicals than cells grown in culture, indicating that growth *in planta* did not increase the tolerance of plant-pathogenic Ralstonia for all stresses (Fig. 6B).

If growth in the plant protects cells from desiccation, this could affect disposal recommendations. To determine if growth *in planta* altered the desiccation tolerance of these bacteria, we measured the survival of plant-pathogenic Ralstonia cells in diseased plant material by thoroughly drying infected tomato stems in a commercial food dehydrator at 35°C. While not strictly equivalent to the 28°C drying that killed all cultured Ralstonia cells, this method ensured uniform and complete desiccation of plant matter that otherwise reabsorbed moisture from the air as the ambient humidity fluctuated. Two of 121 infected stem samples tested contained detectable surviving cells after 12 h at 35°C, a temperature that does not inhibit plant-pathogenic Ralstonia cells growing in culture. Plant-pathogenic Ralstonia are known to survive for extended periods in soil (5). To determine if exposure to the complex but nonplant soil environment could also increase bacterial desiccation tolerance, we measured pathogen survival following complete desiccation of highly infested soil from pots that contained diseased plants. This dried soil contained no detectable plant-pathogenic Ralstonia cells, suggesting that this pathogen’s tolerance of heat and desiccation is increased when the cells are conditioned by growth in plant tissue but not by growth in soil.

An opportunity arose to apply some of these approaches in the context of an export tomato production facility in the tropics. After determining that the farm’s bacterial wilt outbreak was likely caused by R. solanacearum in the irrigation water (see Materials and Methods), we recommended eradicating the pathogen by treating the water with 25 ppm Huwa-San and an in-line UV irradiation system. These interventions drastically reduced bacterial wilt disease incidence in this farm (confidential personal communication from farm manager). Consistent with this outcome, enrichment culture of concentrated treated water samples from the farm did not detect any R. solanacearum cells in 6 quarterly tests over 18 months. In contrast, R. solanacearum was detected in samples of untreated water from a nearby river used as a positive control. This observation suggests that the eradication methods we have validated in the laboratory will also be effective in a field setting.

### Inactivation protocols for downstream applications.

Diagnostic tests, cloning, and quantifying gene expression in R3bv2 all require purified DNA or RNA. To comply with federal regulations that require select agents to be killed with a validated inactivation protocol prior to downstream applications, we measured R. solanacearum survival following four nucleic acid extraction protocols. Representative R3bv2 strain UW551 was cultured as described above and then subjected to the cell lysis step of three different commercial nucleic acid extraction kits according to the manufacturer’s instructions or to the lysis step of a phenol-chloroform extraction protocol. Lysate was then serially dilution plated to quantify any surviving cells, followed by enrichment culture to detect any surviving bacteria below the limit of detection. The purified nucleic acid was also spread on rich Casamino Acids-peptone-glucose (CPG) agar plates and incubated at 28°C for 48 h. No surviving cells were detected in any of these instances, indicating that these four protocols inactivate R. solanacearum R3bv2.

## DISCUSSION

Our goal was to identify chemical and physical treatments that reliably inactivate plant-pathogenic Ralstonia cells, including strains from the highly regulated select agent subgroup. Overall, we found that cultured Ralstonia cells are highly sensitive to heat, UV irradiation, oxidative stress, and desiccation (Table 2).

**TABLE 2:** Summary of treatments that eradicated plant-pathogenic Ralstonia solanacearum cells

Strain	LD_100_ or no. of technical replicates showing survival/total no. of technical replicates tested for indicated treatment									
	Cells suspended in water^*a*^				In biological system^*b*^			On surface^*c*^		
	H_2_O_2_ (ppm)	Huwa-San (ppm)	Bleach (μM)	Heat (°C)	*In planta* heat (°C)^*d*^	Desiccation		Desiccation		UV (exposure time [s])^*i*^
						*In planta* ^*e*^	In soil^*f*^	In tubes^*g*^	On paper^*h*^	
UW24	400	50	120	50	121	0/14	NT	0/3	0/9	20
UW365	200	50	120	50	121	0/13	NT	0/3	0/9	20
UW491	200	12.5	120	50	121	0/8	NT	0/3	0/9	30
UW551	400	25	120	50	121	1/28	0/6	0/3	0/9	20
UW763	400	25	120	50	121	1/19	NT	0/3	0/9	20
UW764	200	25	120	50	121	0/17	NT	0/3	0/9	20
UW770	200	12.5	120	50	121	0/22	NT	0/3	0/9	20

a Cells from overnight cultures were washed and resuspended at 8 × 10^10^ CFU/mL.

b Tomato plants were inoculated with designated RSSC strain by soil drenching (see Materials and Methods).

c LD_100_ following treatment on indicated common laboratory surface.

d Diseased plant material was treated at various temperatures (121°C achieved by autoclaving) and survival of cells quantified as described in Materials and Methods.

e Diseased plant material was dehydrated at 35°C for 12 h and cells quantified as described in Materials and Methods.

f Soil from pots of diseased plants was confirmed to contain living cells and then dehydrated at 35°C for 12 h, and cells quantified as described in Materials and Methods. NT, not tested.

g Cells suspended in water were allowed to desiccate for 120 h at 28°C in open microcentrifuge tubes. Survival is represented here as the number of tube replicates that harbored culturable cells after the treatment period.

h Cells suspended in water were dropped onto the fomites PVC and paper towel and allowed to desiccate for 120 h at 28°C and surviving cells quantified as described in Materials and Methods. Survival is represented here as the number of fomite replicates that harbored culturable cells after the treatment period.

i UV light intensity was 0.2 μmol/m^2^/s, as described in Materials and Methods.

For the purposes of this study, inactivation was defined as leaving no surviving culturable cells. R. solanacearum has been reported to enter a viable-but-not-culturable (VBNC) state as determined by live-dead staining that detects an intact membrane (14, 15). Although there are reports of resuscitation of VBNC Ralstonia cells to culturability, this has been difficult to replicate. Thus, it remains controversial whether VBNC Ralstonia cells pose a risk to growers.

Plant-pathogenic Ralstonia cells survive well in surface waterways, which can be a source of inoculum when they are used for irrigation in agricultural operations (16). Chemical treatments like hydrogen peroxide and bleach are widely used to reduce biological contamination in water (17–19). However, chlorine-containing disinfectants can produce toxic by-products (20, 21). Hydrogen peroxide is not commonly used in agriculture because it degrades rapidly, can damage infrastructure, and is phytotoxic at high concentrations (22). Huwa-San is an appealing alternative because stabilizing hydrogen peroxide on colloidal silver slows its degradation, making it effective at lower concentrations (23). While our experimentally determined LD_100_ of 50 ppm differs from the 20-ppm dosage recommended by the manufacturer, we tested much denser bacterial suspensions than those tested previously (10^10^ versus 10^6^), and we treated for a shorter time (10 min versus 120 min) (18). Consistent with previous observations, we observed that the LD_100_ of each chemical treatment was affected by bacterial cell density, with lower cell densities requiring lower treatment concentrations. When we tested more environmentally relevant bacterial densities of ~10^4^ CFU/mL, all tested dosages described above were effective on these smaller bacterial populations (data not shown).

Physical treatments are used in food handling and processing to eliminate pathogens (24–28). Physical treatments can decontaminate while meeting organic standards, which often preclude chemical treatment (29). Though concerns about resistance selection are less common for physical treatments than for chemical treatments, the mechanism of action of physical treatment is often not fully understood. We tested the efficacy of three physical treatments: heat, desiccation, and UV irradiation.

We found that plant-pathogenic Ralstonia cells cultured in rich medium were completely intolerant of even brief (10 min) exposure to temperatures over 50°C, meaning that heat treatment is a reliable way to eradicate laboratory-grown cells of this pathogen. However, growers and regulators must dispose of plant material, not cultured bacteria. Unfortunately, growth in a plant host dramatically increased the heat tolerance of plant-pathogenic Ralstonia cells, which survived for at least 18 days inside diseased plant material at 50°C (data not shown).

Diseased plants represent a release risk, and agricultural operations rarely have the infrastructure to autoclave or incinerate large amounts of plant material. It is unclear whether plant-pathogenic Ralstonia cells liberated from plant material via decomposition would maintain an increased heat tolerance. Measuring the survival of the pathogen in an agriculturally relevant environment, such as hot compost piles, could answer this question.

Plant-pathogenic Ralstonia cells form biofilms in host plant xylem vessels. These robust biological matrices are linked to the pathogen’s fitness and virulence (12). If the increased heat tolerance of Ralstonia growing *in planta* is due to protection by the biofilm or some other physical or chemical condition in the plant itself, cultured cells and planktonic (nonbiofilm) cells from plants should be similarly sensitive to heat. We observed limited survival of planktonic cells extracted from plants before treatment at 51°C or 54°C. However, planktonic cells that were first heat treated inside plant stems and then extracted and evaluated for survival had much higher survival rates. This supports the hypothesis that being inside a tomato stem makes R. solanacearum more heat tolerant. To probe the question of whether the bacterium must be host conditioned to be more stress tolerant, we also measured the survival of heat-treated R. solanacearum cells in *ex vivo* biofilms on glass slides. If bacteria in biofilms are protected from stress, biofilms grown *ex planta* should also offer protection. Indeed, R. solanacearum cells in biofilms on glass slides did survive 50°C heat treatment for 10 min. This supports the hypothesis that R. solanacearum cells in biofilms are physically or physiologically protected from stress. This is consistent with observations that growth in biofilms increases the stress tolerance of many bacteria (30).

High-energy UV light is commonly used in clinical settings to eliminate pathogen transmission on surfaces (31). This approach is also used to decontaminate agricultural equipment and the surfaces of plants during growth and foods after harvest (32–34). UV light is also a common water decontamination method, for application at both small scales (personal water filters) and large scales (irrigation water treatment) (35). Because plant-pathogenic Ralstonia can be transmitted and dispersed through contaminated equipment, we tested the eradication efficacy of UV light by treating cells on agar plates. This proved to be highly effective. However, we did observe that any cells that were protected from exposure (e.g., stabbed into the agar surface with a pipette tip) survived irradiation. This suggests that direct exposure to UV light is necessary to decontaminate equipment. Furthermore, host-conditioned R. solanacearum cells from stems of infected tomato plants were much more tolerant of UV radiation. Similarly resistant bacteria have been observed in clinical pathogens under antibiotic pressure (8). These findings represent an avenue of future study. Unfortunately, implementation of physical treatment methods on a large scale requires costly infrastructure modifications, such as in-line UV treatment of irrigation water (discussed below). However, the advent of small, portable, and highly efficient light-emitting diode (LED) and visible spectrum irradiation equipment may make this eradication method more accessible to small operations (34, 36).

To demonstrate practical utility of these eradication methods, we tested their effectiveness in the laboratory on three RSSC phylotype I strains isolated from sites currently experiencing bacterial wilt outbreaks. A fortuitous case study allowed us to field test the effectiveness of some of our tested eradication methods on a large export tomato farm in Senegal that was suffering serious yield losses to bacterial wilt disease. The outcome indicates that the treatments described here can be effective under tropical commercial farming conditions.

We also showed that R. solanacearum R3bv2 cells were killed by the lysis step of four different nucleic acid extraction protocols. These validated inactivation protocols satisfy the CDC-USDA requirement that SA research laboratories experimentally verify that whole-agent or subcellular components are fully inactivated (killed) before being removed from registered SA research laboratory space (7). This facilitates downstream biochemical applications, such as DNA-based diagnostic tests or analysis of proteins or gene expression. The cell lysis step of other extraction protocols would probably also inactivate R. solanacearum, but this would need to be experimentally verified using the procedures described here.

We observed minor or no differences among our set of seven plant-pathogenic Ralstonia strains with respect to the LD_100_ for any eradication method tested (Table 2). To explore possible variations in susceptibility among the highly regulated R3bv2 subgroup, we quantified the survival of four different R3bv2 strains. There was no evidence that members of the R3bv2 subgroup were more difficult to eradicate than other plant-pathogenic Ralstonia strains. This means the same practices can be safely used to manage bacterial wilt regardless of whether the subclassification of the infesting strain is known.

Practical and scalable methods to control plant-pathogenic Ralstonia are needed to increase the security of high-value export and subsistence crops. The eradication protocols validated here can be used by growers to control infestations and reduce crop losses to bacterial wilt. They will also support regulators charged with developing phytosanitation and biosafety protocols and researchers who must comply with regulations and prevent the accidental release of quarantine Ralstonia strains.

## MATERIALS AND METHODS

### Strain selection.

The strains used in these studies are described in Table 3. The RSSC has been subdivided into four phylotypes and 3 genomospecies (37, 38). Four R3bv2 R. solanacearum strains isolated from diverse geographical locations were chosen to represent the SA subgroup. Three additional phylotype I plant-pathogenic Ralstonia strains were isolated from plant material from agricultural operations experiencing bacterial wilt outbreaks.

**TABLE 3:** Characteristics of plant-pathogenic Ralstonia strains used in this research

Subgroup	UW strain name	Alternate designation(s)	Phyl-Seq^*a*^	Host	Location	Yr or reference
Race 3 biovar 2 (R3bv2), select agent	UW24	K57	II-1	Potato	Israel	1951
	UW365	POUB (POPS 2), RUN2013	II-1	Potato	China	~1980
	UW491	CIP295, NCPPB2882, RUN2014	II-2	Potato	Colombia	1950
	UW551		II-1	Geranium	Kenya	1999
	UW551-rif	Spontaneous rifampin-resistant variant of UW551	II-1	Geranium	Kenya	42
Non-R3bv2	UW763		I-14	Tomato	Senegal	2017
	UW764		I-18	Rose	Ukraine	2017
	UW770	Rs4001, PD7123	I-18	Rose	Netherlands	2015

a Phylotype and sequevar; see Fegan and Prior (3).

### Preparation of cultured bacterial cells for treatment.

Bacterial strains were cultured from water stocks on Casamino Acids-peptone-glucose plates amended with tetrazolium chloride (CPG+TZC) agar (plus 50 mg/L rifampicin for UW551-rif) for 48 h at 28°C (39). Individual colonies were used to inoculate CPG broth, which was incubated overnight at 28°C with shaking at 225 rpm. After 24 h, cells were pelleted at 6,800 × *g* relative centrifugal force (RCF) and resuspended in sterile water at an optical density at 600 nm (OD_600_) of 0.8 (~1 × 10^10^ CFU/mL). This high-density bacterial cell suspension was then subjected to treatments as described below.

### Quantifying the survival of plant-pathogenic *Ralstonia*.

**(i) Dilution plating.** Treated cell suspensions were serially 10-fold diluted and plated in triplicate on CPG+TZC agar plates (plus rifampicin for UW551-rif) using the drop-dilution plating method (40) and incubated at 28°C for 48 h, and colonies were counted for dilutions showing 3 to 30 colonies to determine the CFU/mL in the original suspension. The average of this triplicate plating was considered a single technical replicate. This method of dilution plating has a limit of detection of 100 CFU/mL.

**(ii) Enrichment culture.** To determine if treated samples contained viable cells below the limit of detection for dilution plating, the remainder of each treated sample was enrichment cultured overnight at 28°C with 225 rpm shaking in 5 mL of CPG broth or, for soil or plant samples, in selective medium South Africa (SMSA) semiselective medium (41). If culture growth was visible, 500 μL of this culture was centrifuged at 6,000 rpm for 5 min in a 1.5-mL microcentrifuge tube. To determine if the growth contained plant-pathogenic Ralstonia cells, the pellet was tested with the Agdia Rs ImmunoStrip test (catalogue no. ISK 33900; Agdia, Inc., Elkhart, IN) according to the manufacturer’s instructions. The limit of detection following this enrichment culture method is 10 CFU/mL (11).

### Preparation of bacterial cells from plants and biofilms.

Unwounded 21-day old tomato plants (bacterial wilt-susceptible cultivar ‘Bonny Best’), grown in 4-inch pots at 28°C with a 12-h light/dark cycle, were inoculated by soil soak (39). Briefly, 50 mL of a stationary-phase bacterial culture, suspended in water as described above, was poured onto the soil of each pot and symptoms were allowed to develop. To assess the behavior of plant-pathogenic Ralstonia cells growing *in planta*, two 0.25-g stem sections were collected from tomato plants at disease index 1 to 2 (1 to 50% of leaves showing bacterial wilt symptoms), with the stem sampled between the cotyledons and the first true leaf.

**(i) Planktonic-cell extraction from infected plant tissue.** Stem sections from infected plants were placed vertically in a 1.5-mL microcentrifuge tube containing 750 μL sterile H_2_O and centrifuged at 17.5 × *g* for 10 min to remove unattached planktonic cells from the xylem vessels and leave behind cells in biofilms. The resulting cell pellets were vortexed to resuspend cells, and the suspensions were subjected to eradication treatments and bacterial survival quantified as described above.

**(ii) Growth from biofilms on glass slides.** Plant-pathogenic Ralstonia cells were cultured as described above and suspended in liquid CPG medium at an OD_600_ of 0.1 (~1 × 10^7^ CFU/mL). This suspension was then used to inoculate glass well chamber slides, as described previously (11), to a final concentration of 1 × 10^6^ CFU/mL. Briefly, inoculated slides were incubated statically at 28°C for 72 h, with the medium decanted and replaced every 24 h. The slides were then dipped in sterile water to rinse away unattached cells. As a control to confirm the presence of a biofilm, a subset of rinsed slides was stained with crystal violet and evaluated microscopically. Experimental slides were placed in 50°C CPG medium for 10 min and then immediately transferred to a room temperature bath to cool. These were then subjected to enrichment culture, and bacterial survival was qualitatively assessed as described above. For UW763, three biological replicates with three technical replicates each were performed (total *n* = 9). For UW551, one biological replicate with three technical replicates each was performed (total *n* = 3). Strain UW551 formed little biofilm on glass slides under these conditions.

### Eradication treatments.

** (i) Chemical treatments.** Hydrogen peroxide (laboratory-grade 30% [vol/vol] H_2_O_2_ solution, catalogue no. 5240-02; Avantor), Huwa-San stabilized peroxide (50% solution; Roam Technology, Ghent, Belgium), and commercial bleach (8.25% sodium hypochlorite, catalogue no. 30966; Clorox) were added to 5-mL suspensions of Ralstonia cells to achieve the desired concentration, mixed thoroughly by vortexing, and allowed to stand at room temperature for 10 min. Cell survival was then quantified by serial dilution plating as described above. For each strain, a total of three biological replicates with three (H_2_O_2_ and Huwa-San) or one (bleach) technical replicates each (total *n* = 3 to 9) was performed.

**(ii) Heat.** Amounts of 100 μL of Ralstonia cell suspensions were transferred to 0.2-mL PCR tubes and incubated at various temperatures in an MJ Research PTC-200 gradient thermal cycler for various times. Cell survival was then quantified by serial dilution plating as described above. For each strain, three biological replicates were performed, with one technical replicate each (total *n* = 3).

**(iii) *In planta* heat.** Infected tomato stem samples obtained as described above were cut into 0.1-g stem sections, placed in a 2.0-mL homogenizer tube with 900 μL sterile water and four stainless steel beads, and ground in a Qiagen PowerLyzer 24 at 2,200 rpm for 1.5 min with a 4-min rest for 2 cycles. The homogenates were then exposed to heat or room temperature and serially dilution plated as described above to determine CFU/g stem. For each strain, three biological replicates were performed, with a minimum of 5 technical replicates.

**(iv) *In planta* desiccation.** Infected tomato stem samples obtained as described above were desiccated in a commercial food dryer (Magic Mill MFD-9050) for 12 h at 35°C. Survival of plant-pathogenic Ralstonia was assessed qualitatively by enrichment culturing the desiccated plant stem in 5 mL SMSA semiselective medium as described above. For each strain, two to three biological replicates were performed, with various numbers of technical replicates (total *n* = 8 to 28).

**(v) In-soil desiccation.** Plants inoculated with UW551-rif were allowed to wilt and die in the pot, releasing plant-pathogenic Ralstonia cells from the roots. A 1- to 2-g soil sample was removed from each pot, and 0.1 to 0.2 g of this soil was cultured overnight at 28°C in SMSA+rif broth and qualitatively assessed for UW551 growth with an Agdia Rs ImmunoStrip to establish an initial plus-or-minus baseline for survival. The remaining soil sample was then dried as described above. Survival was determined qualitatively by enrichment culture of approximately 0.1 to 0.2 g soil in 5 mL SMSA semiselective medium as described above. Three biological replicates were performed, with two technical replicates each. The spontaneous rifampicin-resistant strain UW551 (42) mutant, previously determined to have wild-type virulence and *in planta* fitness, was used to increase selectivity from the dense soil microbiome.

**(vi) Desiccation in tubes.** Amounts of 0.5 mL of bacterial cell suspensions were transferred to 1.5-mL polyethylene microcentrifuge tubes and allowed to stand (lid open) at 28°C for 120 h. Amounts of 0.5 mL sterile water were added to the tubes, and plant-pathogenic Ralstonia survival was quantified by serial dilution plating as described below. For each strain, a total of three biological replicates were performed, with one technical replicate each (total *n* = 3).

**(vii) Desiccation on laboratory surfaces (also known as fomites).** Common laboratory paper towels, chosen because they are used to contain spills, were tested for the ability to harbor live cells after desiccation. Amounts of 100 μL of cell suspensions were placed on brown paper towels sterilized by autoclaving (catalogue no. CS1751; Schilling Supply Company) and allowed to stand in lidded but slightly open sterile petri dishes at 28°C for 120 h. The paper towels were then used to inoculate 5-mL amounts of liquid CPG medium as described above in enrichment culture. For each strain, a total of three biological replicates were performed, with three technical replicates each (total *n* = 9).

**(viii) UV light.** Cell suspensions of plant-pathogenic Ralstonia were serially diluted onto solid CPG medium plates, and the liquid was allowed to absorb completely into the agar. Uncovered plates were then exposed to 0.2 μmol/m^2^/s of radiation (254-nM wavelength) delivered by the germicidal UV lamp of a Baker SterilGard III advance biological safety cabinet (the UV radiation intensity delivered was measured using a UV meter [Apogee Technologies MQ-200]). For each strain, a total of three biological replicates were performed, with one technical replicate each (total *n* = 3).

### Select agent inactivation treatments.

We tested the ability of four nucleic acid extraction protocols to inactivate plant-pathogenic Ralstonia cells as required for compliance with select agent regulations. Briefly, cells of R3bv2 strain UW551 suspended as described above were subjected to the inactivation (cell lysis) step of the following protocols. (i) For the Qiagen RNeasy RNA extraction kit (catalogue no. 74104), the pellet from a 0.5-mL cell suspension was resuspended in 450 μL buffer RLT according to the manufacturer’s instructions. (ii) For the Epicentre MasterPure DNA extraction kit (catalogue no. MC85200), the pellet from a 0.5-mL cell suspension was resuspended in 400 μL C&T lysis buffer according to the manufacturer’s instructions. (iii) For Promega Maxwell DNA extraction, the RSC cultured cells DNA cartridge (catalogue no. AS1620) was used according to the manufacturer’s instructions, with cell viability determined 100 s after bacteria were added to the cartridge. (iv) For the phenol-chloroform extraction method, the pellet from a 0.5-mL cell suspension was resuspended in 5% water-saturated phenol–95% ethanol stop lysis solution. Following these cell lysis steps, R. solanacearum survival was quantified as described above. Inactivation treatments were performed only on R3bv2 strain UW551, and a total of three biological replicates were performed with one technical replicate each (total *n* = 3).

### Experimental design and data analysis.

Each experiment contained multiple biological and/or technical replicates. For in-culture assays, each biological replicate consisted of a single overnight culture which was inoculated with a single isolated colony from an agar plate. Technical replicates were repeats of each experiment using the same starting culture and suspension (i.e., one culture divided into three suspensions was one biological replicate with three technical replicates). For *in planta* experiments, one biological replicate consisted of all plants treated with inoculum from an overnight culture grown from a single colony as described above. One technical replicate was a single plant inoculated with that starting culture (i.e., one culture used to generate inoculum for 15 plants was one biological replicate with 15 technical replicates). Data were analyzed using Prism software. For all experiments, the Mann-Whitney test was performed to compare the untreated control group (all replicates from all strains) and the first treatment (i.e., lowest concentration, temperature, or time exposure as indicated in figure captions) to show no surviving cells of any strain. The resulting *P* values are indicated in the relevant figure legends.

### Application of disinfestation strategies in a field setting.

The practical use of two combined disinfestation methods was informally assessed in collaboration with a large commercial tomato producer in Senegal suffering a widespread outbreak of bacterial wilt. R. solanacearum strain UW763 (phylotype I, sequevar 14; whole-genome sequence at NCBI Bioproject number PRJNA591018 [38]) was isolated from the surface-sterilized stem of a symptomatic tomato plant from this production facility. The farm’s irrigation system and water from the nearby river that was used as an irrigation source contained very similar R. solanacearum strains, as determined by the sequences of the diagnostic *egl* gene. This suggested that contaminated irrigation water was the source of the outbreak. The farm’s irrigation water was subsequently treated with 25 ppm Huwa-San and an in-line UV irradiation system was installed. Following implementation of this treatment regime, we tested three or more 1.5-L samples of treated irrigation water from this facility every 3 months for the presence of R. solanacearum using enrichment culture as described above. Briefly, water samples were centrifuged at 6,000 rpm for 20 min to pellet any bacteria. After supernatants were decanted, pellets were resuspended in 5 mL SMSA broth, incubated at 28°C with shaking for 48 h, and tested for the presence of R. solanacearum using Agdia Rs ImmunoStrips according to the manufacturer’s instructions.
